# Minimally invasive surgery guidelines in paediatric surgical oncology - role of MIS in fertility preservation

**DOI:** 10.3332/ecancer.2025.2028

**Published:** 2025-11-13

**Authors:** Marianna Cornet, Julien Grosman, Aurore Pire, Sabine Sarnacki

**Affiliations:** Department of Paediatric Surgery, Urology and Transplantation, Necker Enfants-Malades Hospital, AP-HP, University Paris Cité, Paris 75015, France

**Keywords:** fertility preservation, minimally invasive surgery, gonadotoxicity, ovarian sparing surgery, ovarian transposition, uterine transposition, cryopreservation

## Abstract

Childhood cancers represent approximately 1% of all malignancies, with improved therapeutic strategies leading to an 80% long-term survival rate. However, these advancements come with potential long-term sequelae, among which fertility impairment is a major concern. Gonadotoxic treatments, including chemotherapy, radiotherapy and mutilating surgery, significantly impact reproductive potential, necessitating fertility preservation strategies. Minimally invasive surgery (MIS) plays a crucial role in preserving fertility in paediatric patients. Ovarian and genital tract-sparing surgery should be prioritised for benign ovarian tumours, which constitute 90% of childhood ovarian lesions, to avoid unnecessary loss of ovarian reserve. Ovarian transposition is recommended for patients requiring pelvic radiotherapy, relocating the ovaries outside the radiation field to mitigate ovarian damage. Additionally, uterine transposition has been explored to protect reproductive organs from radiation exposure. Ovarian tissue cryopreservation remains a promising option, particularly for prepubertal patients undergoing gonadotoxic treatments. Cryopreserved ovarian fragments can later be used for autografting or *in vitro* maturation, though the risk of malignant cell transmission remains a challenge. MIS contraindications are limited, primarily related to tumour size and the risk of rupture during laparoscopic procedures. A multidisciplinary approach involving oncologists, surgeons, radiotherapists and fertility specialists is essential for optimising outcomes. This chapter discusses the indications, techniques and challenges associated with MIS in fertility preservation, emphasising its role in ensuring reproductive potential while maintaining oncological safety in paediatric cancer patients.

## Introduction

Childhood cancers currently account for approximately 1% of all malignancies. Advances in the understanding of paediatric cancer biology have significantly improved therapeutic approaches, leading to an 80% long-term overall survival rate, primarily through intensified targeted chemotherapy and/or radiotherapy [1]. However, these advances come at the cost of potential long-term adverse effects [2].

Among these, fertility impairment is a major concern, representing one of the most significant long-term sequelae of paediatric cancer treatment. It carries the risk of reduced reproductive potential and can have profound psychological repercussions. Fertility disorders may result from gonadotoxic treatments, such as chemotherapy and radiotherapy, as well as from mutilating surgical procedures affecting reproductive organs [3].

The paediatric oncologic surgeon, therefore, plays a crucial role in fertility preservation for patients undergoing cancer treatment. The goal is to minimise long-term sequelae in these young patients who have their whole lives ahead of them. In this context, minimally invasive surgery (MIS) is essential, as it helps reduce visible scarring and ensures a faster recovery, which is particularly important to prevent delays in treatment initiation. Ensuring timely access to treatment is critical for optimising outcomes, allowing patients to start their therapies without unnecessary postponements.

### Mechanisms of induced infertility

#### In Females

Alkylating agents, such as cyclophosphamide, which are widely used—particularly in high-dose conditioning chemotherapy—are among the most gonadotoxic and frequently lead to ovarian failure.

Ionising radiation affects both dividing and non-dividing cells. As with chemotherapy, the impact of radiotherapy on ovarian function depends on patient age, radiation field, daily dose and cumulative dose received. Permanent ovarian failure has been associated with radiation doses of 10–20 Gy in children and 4–6 Gy in adults. Moreover, uterine irradiation during childhood is known to increase the risk of preterm birth and low birth weight offspring [1, 4, 5].

Dose fractionation and intensity-modulated radiation therapy in external beam radiation therapy (EBRT) have been associated with reduced ovarian damage due to the potential for cellular repair between fractions [6, 7]. Pelvic brachytherapy, which is used mostly for urogenital rhabdomyosarcomas (RMS) and other sarcomas, is expected to be less toxic than EBRT but may lead to vaginal stenosis, which can impair fertility [8]. Additionally, irradiation of the hypothalamic-pituitary-adrenal (HPA) axis at doses ≥30 Gy can result in gonadotropic failure, although ovarian reserve is generally preserved [9].

Finally, mutilating surgery, partial resection of the genital tract and/or ovaries or even any kind of pelvic surgery, especially since it is associated with irradiation, may impair fertility potential.

#### In Males

Prepubertal gonads are highly susceptible to the gonadotoxic effects of chemotherapy administered during childhood. Alkylating agents can impair spermatogenesis, though testosterone secretion is usually preserved.

In contrast, radiation therapy affects both spermatogenesis and testosterone production. Spermatogonia are sensitive to doses as low as 0.15 Gy and are completely eradicated at doses ≥2 Gy. Prepubertal males are more vulnerable to the effects of irradiation on spermatogenesis and Leydig cell function compared to adolescents and adults. Notably, unlike females, fractionated radiation doses induce higher gonadal toxicity than a single exposure.

Irradiation of the HPA axis at doses ≥25–30 Gy can cause hypogonadotropic hypogonadism, leading to pubertal delay, sexual dysfunction and infertility, with a dose-dependent effect [10]. Childhood cancer survivors show a 50% decrease in testicular descent compared to the general population [11]. Additionally, long-term follow-up studies have reported a significant increase in fertility disorders (46% in survivors versus 17.5% in siblings; RR = 2.64, 95% CI 1.88–3.70, *p* < 0.001) with documented sperm abnormalities in a cohort of 1,600 childhood cancer survivors [12].

Regarding fertility preservation in males, MIS does not have a role; indeed, testicular cryopreservation, testicular transposition or testicular sparing surgery are performed exclusively through an open surgical approach. In this chapter, we will present the various aspects of MIS for fertility preservation in females paediatric patients undergoing cancer treatment.

## MIS indications

### Ovarian and genital tract sparing surgery

In girls, ovarian sparing surgery is mandatory to preserve fertility in case of benign ovarian tumours, which represent around 90% of childhood ovarian lesions (benign germ cell or benign epithelial tumours). Indeed, 13% of ovarian teratomas are bilateral (either synchronous or metachronous) [13] and unilateral ovariectomy leads to depletion of the total number of primordial follicles and, therefore, might increase the risk of premature or early menopause, although not clearly demonstrated in children. If in adults evidence indicates that having a single ovary does not reduce the fertility potential (with normal live birth rates, spontaneously or after assisted reproductive techniques), evidence from long-term studies in children is missing [14, 15]. In the absence of evidence in the paediatric population, ovarian sparing surgery should always be considered.

In everyday practice, deciding whether to spare the ovary because the lesion seems benign or to perform a total ovariectomy because signs of malignancy are present is not always straightforward [16]. Two different situations should be considered:

1. In asymptomatic (painless) girls, the surgeon has enough time to perform reliable imaging (ensuring that the tumour does not show any sign of malignancy) and to dose tumour markers alpha-fetoprotein, human chorionic gonadotrophin, Inhibin B, anti-Mullerian hormone and Calcemia. In these situations, laparoscopic exploration is recommended as the first step of the procedure. The primary goal of laparoscopy is to better appreciate the location and nature of the lesion, and in malignant cases, to precisely stage the disease. However, laparoscopic ovarian tumour resection is not recommended by the present authors and laparotomy via a supra-pubic approach remains the preferred method. The rationale for open surgery in paediatric ovarian tumours includes the potential presence of malignancy even in cystic lesions (e.g., rare cases of juvenile granulosa cell tumours) or those appearing as mature teratomas, which may contain both mature and immature components, where intraoperative rupture would significantly worsen prognosis. Additionally, the open approach remains the safer option for maximising the preservation of healthy ovarian tissue when ovarian-sparing surgery is chosen for a tumour with all the characteristics of a benign lesion. The wish to favour aesthetic considerations in the treatment of ovarian masses in children could then lead to a loss of oncological control, which seems unacceptable considering that the classic treatment proposes a supra-pubic approach that results in the same post-operative course.
2. When the patient is complaining of acute pain due to adnexal torsion or ovarian rupture, the best approach is to perform a laparoscopic exploration in an emergency. If ovarian rupture is diagnosed, peritoneal inspection, sampling of ascites are the rule. Complete ovariectomy or adnexectomy in the same operative time should be discussed only if the ovary and/or the peritoneum indicate malignant features. If adnexal torsion is seen, detorsion is recommended as it allows to approach the potential underlying diagnosis (benign or malignant tumour) with imaging and markers dosage in the post-operative period.

Preservation of the genital tract should be considered when planning surgical interventions for tumours involving these anatomical structures. The various pathological entities encountered include: RMS of the vagina, cervix or uterus; malignant germ cell tumours (GCT) of the vagina; and clear cell adenocarcinoma, which is typically diagnosed later in life. The prognosis of these tumours depends on achieving local tumour control, which is now managed with less radical surgical approaches compared to those employed in previous decades. For urogenital RMS, treatment may be accomplished without surgical intervention when complete remission is achieved following chemotherapy or it may be supplemented with localised brachytherapy to avoid mutilating surgery, the latter of which is now exceptionally rare [17, 18]. In cases of vaginal GCT, surgical resection of the primary tumour site remains essential; however, the efficacy of neoadjuvant chemotherapy often precludes the need for mutilating surgery, enabling a more conservative approach such as partial trachelectomy.

### Ovarian transposition

Ovarian transposition was the first procedure proposed to preserve fertility in girls with cancer and is indicated for patients with tumours requiring pelvic radiation of 42–58.4 Gy, much higher doses than those that can induce loss of ovarian function (4–20 Gy) [5]. The ovaries are very radiosensitive organs. The extent of damage depends on the dose of radiotherapy that reaches the ovaries, the age of the girl at the time of treatment and the associated drugs used for chemotherapy. Doses of 10–20 Gy in children and 4–6 Gy in adults are associated with permanent ovarian failure [19].

Preliminary ovarian transposition should be considered for all children with tumours requiring radiotherapy implicating part of the pelvis. RMS of the bladder, vagina or uterus or soft tissue or pelvic bone sarcomas, such as Ewing’s sarcoma, are the main indications for ovarian transposition in children [5, 6, 20].

Ovarian transposition is designed to place the ovaries outside of the irradiation field. Detailed analysis of the initial pelvic MRI should be done to ensure that the tumour does not involve the ovarian region.

The planning target volume should be precisely defined by the surgeon and the radiotherapist before ovarian transposition.

### Uterine transposition

In some cases, radiation is an important complementary treatment. But the area can involve the pelvic region and result in a deleterious effect to fertility, which may affect the uterus and ovaries. The uterine and the adnexal, transposition to an upper abdominal region during the radiation therapy may protect these organs with security. Some cases are described in adult women [21, 22], and a case in a pre-pubertal girl was described with successful [23].

### Ovarian tissue cryopreservation

Main indications nowadays are represented by myeloablation before bone marrow or stem cell transplantation, total body irradiation and high-dose chemotherapy with alkylating agents [24]. There is some controversy about the amount of ovarian tissue to retrieve, as some teams propose to do a partial ovariectomy or to harvest only the cortex. Since follicular loss can reach up to 65% only due to the ischemia hit [25], it seems for many groups essential to collect an entire ovary in younger children in order to get enough tissue or cells for the future pregnancy project. It has to be noted that in most of the team the ovarian tissue is more used for fertility than for puberty restauration [26, 27].

Cryopreserved ovarian fragments might further be used for either autografting or *in vitro* maturation of primordial follicles. Since Donnez *et al* [28, 29] and Meirow *et al* [30] published the first 2 pregnancies obtained after autograft of ovarian cortex, more than 200 livebirths have been documented in the literature after autologous grafting of previously cryopreserved adult ovarian tissue. The ovarian function is restored after 4 months following grafting and 23%–37% of these women will have pregnancies. In prepubertal patients, results are not assessable, as most of these patients have not achieved the age of parental desire. Poirot *et al* [31] confirmed that induction of puberty was efficient by heterotopic autografts of ovarian fragments. In 2015, Demeestere *et al* [32] reported the first pregnancy after cryopreservation at a paediatric age. The patient, who required myeloablation before bone marrow transplantation for sickle cell anemia, was 14 years old but premenarchal at the age of cryopreservation. She gave birth 13 years later to a normal child, 2 years after bilateral autografting of ovarian fragments and natural conception. Besides this publication, no cohort studies or even isolated cases of livebirths after prepuberty ovarian tissue cryopreservation for malignancy have been reported [33].

A major problem raised by autografting of previously cryopreserved ovary is the risk of reintroduction of the primary disease in the patient’s organism, especially when the primary disease is likely to give metastasis to the gonads, such as leukaemia or lymphoma. Shaw *et al* [34] managed to prove, in mice models, that cryopreserved ovarian tissue samples from donors with lymphoma can transmit cancer to grafted recipients. Recent studies showed that the risk is highest in leukaemia patients, moderate in gastrointestinal cancers and low in breast cancer, sarcomas, gynecological cancers, Hodgkin’s and non-Hodgkin’s lymphomas [35, 36]. A way to avoid this problem will be the *in vitro* culture of primordial follicles harvested from the cryopreserved tissue, a technique which is currently still experimental [37].

## MIS contraindications

There are very few contraindications to MIS concerning fertility preservation. In the case of a large ovarian tumour extending beyond the umbilicus, initiating surgery with an open laparoscopy through the umbilical approach is contraindicated due to the risk of tumour rupture during trocar insertion. It is recommended to perform lesion resection via laparotomy as the first step.

For other fertility preservation techniques, the only contraindication is the presence of extensive adhesions preventing the feasibility of laparoscopy.

## Surgical approach

### Preoperative workup

- Blood tests: Complete blood count and coagulation profile are required. If cryopreservation is planned, viral serologies (HIV, hepatitis B, hepatitis C and TPHA/VDRL) should be obtained.
- Imaging: Pelvic MRI is requested to assess ovarian tumours. In cases of gonadal transposition for radiation protection, collaboration with the radiotherapy team is essential to ensure optimal gonadal repositioning outside the radiation field. For ovarian tissue preservation, imaging is not required.

Given the broad range of fertility preservation strategies, which depend on age, tumour type and treatment plan, multidisciplinary board discussions are highly recommended. These should include medical oncologists, radiation oncologists and fertility preservation specialists to determine the most appropriate approach.

A detailed informed consent should be provided and discussed with the patient and their parents to ensure a comprehensive understanding and shared decision-making.

### Patient position

The patient is positioned supine and in Trendelenburg position, which causes the intestine to fall into the upper part of the abdomen to free the pelvis and enable visualisation of the genital tract. The arms are placed alongside the body. The bladder is emptied with a transurethral Foley catheter [5, 38].

### Trocar sites

An umbilical trocar is inserted either via an infra-umbilical incision or directly through the umbilicus. Carbon dioxide is insufflated at a pressure of 8 to 12 mm Hg, dependent on the patient’s age and weight. Two working trocars (3–5 mm) are inserted under direct vision into the right and left flank, almost as the same level as the umbilicus, depending on the child’s age. The smaller the child, the higher the trocars should be placed on the abdomen to ensure a better working space. A 0° or 30° angled camera can be used.

### Surgical technique

#### Ovarian tumours

As described above, the goal of laparoscopy is to better assess the location and nature of the lesion. The supramesocolic area and anterior parietal peritoneum should be visualised with biopsies taken from any suspicious areas. A sample of ascitic fluid or peritoneal washings should be collected for cytological analysis if no ascites is present. Examination of abdominal organs, particularly the diaphragmatic domes, is essential. Omentectomy is recommended if the omentum, or any part of it, appears abnormal. Pelvic and retroperitoneal lymph nodes should be carefully evaluated, with biopsies of any abnormal nodes. The contralateral ovary should be assessed.

The second step of the procedure involves laparotomy.

If the tumour is large and extends beyond the umbilicus, peritoneal exploration via laparoscopy should be performed after open surgery.

## Ovarian transposition

Various sites for ovarian transposition can be considered. In case of a midline irradiation field (urogenital tumours or medulloblastoma), both ovaries are usually placed away from the midline, laterally in the paracolic gutters or laterally and anteriorly near the inguinal ring (bilateral ovarian transposition). In case of a lateral tumour (RMS or Ewing’s sarcoma), the compromised ovary is placed on the opposite site of the tumour (unilateral ovarian transposition). In some cases of Hodgkin’s lymphoma, when the irradiation field implicates the bilateral iliac chains and inguinal regions, the ovaries are placed in line with the iliac crests (bilateral ovarian transposition) [39].

In most of the cases, it is not necessary to section any ovarian ligament. In prepubertal girls, the ovaries are located higher in the pelvis and the ligaments are more stretchable than in adult women [38]. The blood supply to the ovaries should be carefully preserved, especially the ovarian vessels should be examined to ensure the absence of any kinking or direct injuries [40]. The ovaries are grasped with atraumatic forceps and mobilised above the iliac crest level (in the case of midline irradiation field) as high as possible without any dissection or division of the ovarian ligaments or of the fallopian tube. The ovaries are sutured to the peritoneum with resorbable or non-resorbable suture material and are marked by metallic clips to make them visible on imaging before the initiation of EBRT.

In the case of pelvic brachytherapy, ovarian transposition is only needed for a short time. The ovaries can then be sutured to the anterior abdominal wall by a transfixing stitch of non-resorbable suture material, knotted on the outside of the patient on a pledget. The stitch knotted on the outside holding the ovary to the abdominal wall is removed at the end of brachytherapy, to return the ovaries to their normal position without the need for reoperation [38].

Whatever technique is used, the surgical procedure should be performed as close to the time of radiation treatment as possible, due to the risk of remigration of the ovaries [41].

## Ovarian harvesting for cryopreservation

The ovary is grasped with atraumatic forceps. Hemostasis is performed with bipolar hemostatic forceps, and the mesovarium is cut with scissors. The ovary is placed within a bag and removed via the umbilical trocar. The ovary is then immediately placed in a transport medium and sent to the reproductive biology unit to be cryopreserved.

After isolation and fragmentation, ovarian cortex fragments are slowly frozen in an automated freezer down to the temperature of liquid nitrogen, in which they are stored. Histological analysis of some fragments is mandatory as it allows searching for malignant cells in both cortex and medulla, particularly in tumours with potential ovarian spread (hematological cancer and neuroblastoma) and also to estimate the follicular wealth of the tissue, notably in case of previous gonadotoxic treatment.

### Tips and pitfalls

1. For ovarian tumour surgery, a thorough preoperative assessment is essential. In the case of a malignant tumour, ovarian preservation is not possible. In the case of a benign tumour, caution is required if the tumour is large to avoid rupture during exploratory laparoscopy or during detorsion. To minimise the risk of tumour rupture as much as possible, the authors of this chapter recommend performing laparotomy for ovarian sparing surgery. In large cystic tumours, in cases where a benign tumour is highly suspected, the cyst is first covered with a sterilised surgical sheet applied with quick-drying glue [42] and then punctured using a 5 mm laparoscopic trocar connected to suction. Once the cyst(s) are drained, the ovary can be exteriorised and a suture is placed as the trocar is removed. The cyst resection surgery can then be performed with protection of the operative field.
2. If the tumour is large and extends beyond the umbilicus, peritoneal exploration via laparoscopy should be performed after open surgery.
3. Proper patient positioning is crucial, with the placement of a urinary catheter to ensure that the bladder remains empty throughout the procedure.
4. Regarding ovarian tissue cryopreservation, it is important in prepubertal girls to remove an entire ovary to avoid damaging the ovarian tissue. Atraumatic forceps should be used and the use of energy-based instruments should be minimised to avoid damaging the ovarian tissue.

## Conclusion

Fertility preservation in paediatric oncology has become a crucial aspect of patient care, given the significant gonadotoxic effects of chemotherapy, radiotherapy and certain surgical interventions. MIS plays an essential role in reducing treatment-related morbidity while optimising reproductive outcomes. Techniques such as ovarian and uterine transposition, ovarian sparing surgery and ovarian tissue cryopreservation have shown promising results in protecting future fertility, particularly when applied in a timely and well-coordinated manner.

A multidisciplinary approach involving oncologists, surgeons, reproductive specialists and radiotherapists is essential to tailor fertility preservation strategies to each patient's needs.

Future research should focus on improving fertility preservation techniques, optimising cryopreservation protocols and enhancing post-treatment fertility restoration options. As survival rates in childhood cancer continue to improve, ensuring the quality of life of survivors—including their reproductive potential—should remain a top priority in paediatric oncology.

## Conflicts of interest

No conflicts of interest.

## Funding

No funding declaration.
